# The impact of albendazole treatment on the incidence of viral- and bacterial-induced diarrhea in school children in southern Vietnam: study protocol for a randomized controlled trial

**DOI:** 10.1186/s13063-016-1406-1

**Published:** 2016-06-06

**Authors:** Jacqueline M. Leung, Chau Tran Thi Hong, Nghia Ho Dang Trung, Hoa Nhu Thi, Chau Nguyen Ngoc Minh, Thuy Vu Thi, Dinh Thanh Hong, Dinh Nguyen Huy Man, Sarah C. L. Knowles, Marcel Wolbers, Nhat Le Thanh Hoang, Guy Thwaites, Andrea L. Graham, Stephen Baker

**Affiliations:** Department of Ecology and Evolutionary Biology, Princeton University, Princeton, NJ USA; The Hospital for Tropical Diseases, Wellcome Trust Major Overseas Programme, Oxford University Clinical Research Unit, 764 Vo Van Kiet, Quan 5, Ho Chi Minh City, Vietnam; Department of Infectious Diseases, Pham Ngoc Thach University of Medicine, Ho Chi Minh City, Vietnam; Department of Parasitology and Mycology, Pham Ngoc Thach University of Medicine, Ho Chi Minh City, Vietnam; Cu Chi Preventive Medicine Centre, Ho Chi Minh City, Vietnam; The Hospital for Tropical Diseases, Ho Chi Minh City, Vietnam; Department of Pathology and Pathogen Biology, The Royal Veterinary College, Hertfordshire, UK; Centre for Tropical Medicine, University of Oxford, Oxford, UK; The London School of Hygiene and Tropical Medicine, London, UK

**Keywords:** Albendazole, Deworming, Soil-transmitted helminths, Diarrhea, Co-infection

## Abstract

**Background:**

Anthelmintics are one of the more commonly available classes of drugs to treat infections by parasitic helminths (especially nematodes) in the human intestinal tract. As a result of their cost-effectiveness, mass school-based deworming programs are becoming routine practice in developing countries. However, experimental and clinical evidence suggests that anthelmintic treatments may increase susceptibility to other gastrointestinal infections caused by bacteria, viruses, or protozoa. Hypothesizing that anthelmintics may increase diarrheal infections in treated children, we aim to evaluate the impact of anthelmintics on the incidence of diarrheal disease caused by viral and bacterial pathogens in school children in southern Vietnam.

**Methods/design:**

This is a randomized, double-blinded, placebo-controlled trial to investigate the effects of albendazole treatment versus placebo on the incidence of viral- and bacterial-induced diarrhea in 350 helminth-infected and 350 helminth-uninfected Vietnamese school children aged 6–15 years. Four hundred milligrams of albendazole, or placebo treatment will be administered once every 3 months for 12 months. At the end of 12 months, all participants will receive albendazole treatment. The primary endpoint of this study is the incidence of diarrheal disease assessed by 12 months of weekly active and passive case surveillance. Secondary endpoints include the prevalence and intensities of helminth, viral, and bacterial infections, alterations in host immunity and the gut microbiota with helminth and pathogen clearance, changes in mean *z* scores of body weight indices over time, and the number and severity of adverse events.

**Discussion:**

In order to reduce helminth burdens, anthelmintics are being routinely administered to children in developing countries. However, the effects of anthelmintic treatment on susceptibility to other diseases, including diarrheal pathogens, remain unknown. It is important to monitor for unintended consequences of drug treatments in co-infected populations. In this trial, we will examine how anthelmintic treatment impacts host susceptibility to diarrheal infections, with the aim of informing deworming programs of any indirect effects of mass anthelmintic administrations on co-infecting enteric pathogens.

**Trial registration:**

ClinicalTrials.gov: NCT02597556. Registered on 3 November 2015.

**Electronic supplementary material:**

The online version of this article (doi:10.1186/s13063-016-1406-1) contains supplementary material, which is available to authorized users.

## Background

Soil-transmitted helminths (STH) are a group of intestinal nematodes that can infect humans who come into contact with helminth eggs or larvae in soil, food, or water contaminated with feces. The four main species of human STH are the roundworm, *Ascaris lumbricoides*; the whipworm, *Trichuris trichiura*; and the hookworms, *Necator americanus* and *Ancylostoma duodenale*. Recent estimates suggest that *A. lumbricoides* infects 819 million people, *T. trichiura* 464.6 million people, and hookworms 438.9 million people worldwide, with greatest numbers occurring in sub-Saharan Africa, Asia, and South America [1].

Most infections caused by STH are asymptomatic, especially when few worms are present. Heavy helminth burdens, however, can lead to growth stunting, impaired cognitive development, and malnutrition in infected hosts [2]. When identified, heavily infected individuals should always be dewormed because they are at highest risk of morbidity and are a major source of environmental contamination with helminth eggs [3, 4]. However, only a few hosts harbor such large burdens, while most individuals harbor relatively few STH [5]. In fact, low-intensity helminth infections may actually be beneficial to hosts, as they may protect against detrimental effects of other pathogens [6, 7] and autoimmune diseases [8].

Currently, in an effort to reduce helminth burdens, the World Health Organization (WHO) recommends that single-dose anthelmintics (400 mg albendazole or 500 mg mebendazole) be routinely administered to school children either once a year in areas with STH prevalence between 20 % and 50 % or twice a year in areas with STH prevalence above 50 % [9]. This intervention is aimed to reduce morbidity by reducing worm burden [3, 4]. However, a recent Cochrane review and meta-analysis found almost no evidence of benefits to nutrition, cognitive development, school performance, or survival in 45 mass deworming studies, although there was no differentiation between infected and uninfected individuals in these analyses [10]. Additionally, individuals infected with STH also harbor many other infectious agents, including virulent viral and bacterial pathogens [11, 12], which may be indirectly impacted by anthelmintic treatment, as demonstrated in studies of naturally co-infected animals [13–15]. Current deworming programs focus solely on reducing the morbidity caused by helminth infections and often fail to monitor for indirect consequences of treatment on other pathogens. As human health reflects the integrated impact of all infectious agents to which hosts are exposed, it is crucial to monitor for unintended effects of mass drug treatments in co-infected populations.

Recent experimental and clinical evidence suggests that anthelmintic treatments may actually increase host susceptibility to other gastrointestinal infections (bacteria, viruses, or protozoa) [14–18]. For example, a study by Blackwell et al. in lowland Bolivia found that *Giardia lamblia* infection was less likely in helminth-infected individuals compared to helminth-uninfected individuals and that treatment with anthelmintics marginally increased the odds of future *G. lamblia* infections over the course of 6 years [16]. This finding supports the results of a previous study, which suggested that treatment for helminths may increase susceptibility to *G. lamblia* infections in children in Bangladesh [19]*.* Two studies in wild mice have shown that while a single oral dose of the anthelmintic, ivermectin, significantly reduced nematode infections, it resulted in a simultaneous increase in gastrointestinal coccidial protozoa [14, 15] and cestodes [15]. Additionally, anthelmintic treatment with pyrantel pamoate in free-living yellow-necked mice was shown to increase the abundance of specific ectoparasitic ticks [18]. In each of these cases, anthelmintic treatment resulted in a concomitant increase of another co-infecting parasite species, suggesting that the targeted helminths were suppressing these infections [13]. As most individuals with helminth infections are co-infected with multiple parasite species, understanding the impact of mass deworming programs on non-target pathogens, especially those posing high health risks, is warranted.

The aim of this study is to investigate the effects of anthelmintic treatment on diarrheal diseases caused by intestinal viral and bacterial pathogens in school children in southern Vietnam. High rates of co-infections are expected, due to the overlapping distributions and similar routes of transmission of STH and diarrheal pathogens. The shared intestinal niche between these pathogens also suggests that direct and indirect interactions will occur within the human gut, potentially impacting the disease dynamics of each pathogen in unexpected ways.

The proposed study will be conducted in Vietnam, which has among the highest prevalence levels of human STH of any Western Pacific country [20]. Diarrheal disease also remains a major cause of mortality and morbidity in children in Vietnam, with 11 % of deaths in children aged 0–5 years being due to diarrhea [21]. Recently, the causative agents of diarrheal disease in children under 5 years of age in Ho Chi Minh City, Vietnam, were identified, with Norovirus, Rotavirus, *Campylobacter, Salmonella,* and *Shigella* being the main pathogens isolated from diarrheal patients [22]. Further analysis of a subset of these samples by real-time polymerase chain reaction (PCR) found STH eggs in 6/342 (1.8 %) diarrheal samples, whereas 41/123 (33.3 %) of healthy control samples were positive for STH (our unpublished data). Previous studies have found that real-time PCR is sensitive enough to detect even one STH egg in 200 mg of fecal material [23]. No statistical differences in demographics or personal hygiene between helminth-infected and helminth-uninfected individuals were present, including age, sex, address, household size and income, water and food resources, toilet type, probiotic usage, or hand-washing practice. The significantly higher prevalence of STH infections in healthy controls compared to diarrheal patients suggests that STH may be beneficial to children in combating the pathogens that cause diarrhea. Thus, the primary objective of this study is to investigate the effects of 400 mg albendazole treatment on the incidence of diarrheal disease caused by viral and bacterial pathogens in school children in southern Vietnam. This study will provide new insights into the role of co-infections in the success of treatment strategies and may inform public health policies to mitigate indirect effects of mass deworming programs.

## Methods/design

### Study aims

We hypothesize that anthelmintic treatment with 400 mg albendazole will lead to an increase in the incidence of diarrheal disease caused by viral or bacterial pathogens, potentially due to alterations to the host immune response and/or gut microbiota with helminth removal. To test this hypothesis, we will compare the incidence of diarrheal disease between individuals receiving anthelmintic and placebo treatment in a randomized, double-blinded, placebo-controlled trial over the course of 12 months. Secondarily, this study will determine the prevalence and intensities of helminth, viral, and bacterial infections in the study location, alterations in host immunity and the gut microbiota with helminth and pathogen clearance, changes in mean *z* scores of body weight indices over time, and the number and severity of adverse events (AE). The study design presented herein aims to investigate whether there are any indirect effects of anthelmintic treatment on non-target diarrheal pathogens, with the goal of informing deworming programs in co-infected populations.

### Study area

This study will be conducted in three primary schools in Cu Chi district in Ho Chi Minh City, Vietnam. Cu Chi is a peri-urban district on the northwestern border of Ho Chi Minh City. The climate of Cu Chi district is temperate with two distinct seasons: a rainy season from May to November and a dry season from December to April. Agriculture is the main source of income in the province. In 2010, Cu Chi district had a population of approximately 355,822 people, with a population density of 819 persons/km^2^ [24]. A prior study indicated that hookworm infections are the predominant STH in this area, with most children experiencing low infection intensities [25]. Although parts of Vietnam participate in the national deworming program administered by the WHO, the primary schools involved in this study have not provided deworming medication to their school children since January 2014 due to financial costs of the medication. The schools have no plans to provide anthelmintic medication to school children in the upcoming year.

### Study design

**Fig. 1:**
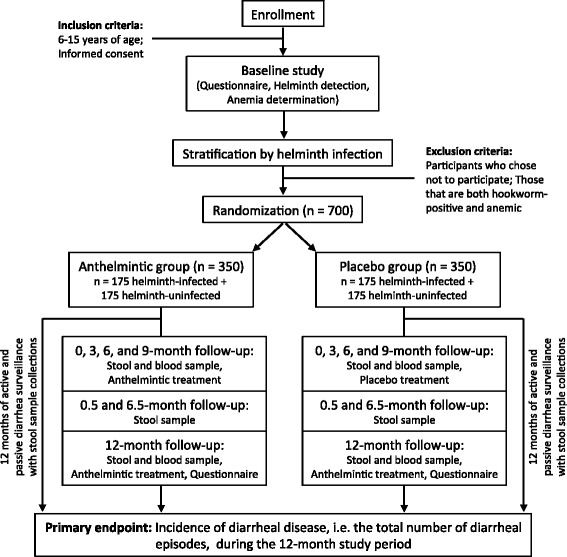
Flowchart of the study

This study is a randomized, double-blinded, placebo-controlled trial designed to investigate the effects of anthelmintic treatment (400 mg albendazole) on the incidence of viral- and bacterial-induced diarrheal disease in school children aged 6–15 years in Cu Chi district in Ho Chi Minh City, Vietnam. This study was designed following the Standard Protocol Items: Recommendations for Interventional Trials (SPIRIT) 2013 statement (Additional file 1) and will have two phases: (1) a baseline study and (2) a clinical trial. The baseline study will assess the current prevalence and intensity of STH infections in approximately 1800 school children in Cu Chi district. A stool sample will be collected for STH detection and quantitation by real-time PCR and microscopy. Hematocrit levels will be assessed from finger-prick blood draws to monitor the risks of anemia with different STH infections. From this baseline study, 350 helminth-infected and 350 helminth-uninfected individuals will be selected from larger groups using a random number generator, then recruited and randomized to receive either anthelmintic or placebo treatment once every 3 months for 12 months. At the end of 12 months, all participants will receive anthelmintic treatment. Weekly active and passive surveillance of diarrheal cases will be conducted. A study nurse will call the parents/guardians of each participant weekly, asking if any incidence of diarrhea has occurred. Participants will also inform the study nurse of diarrheal cases through passive surveillance. Stool and blood samples will also be collected throughout the study for parasitological and immunological analyses (Fig. 1).

### Eligibility

To be eligible for enrollment into the baseline study and clinical trial, participants will need to be between 6 and 15 years of age and have written informed consent from a parent or guardian. Children over 10 years of age will themselves also be required to provide written assent in order participate in this study. This age group was specifically selected because these individuals are expected to have the highest intensity of STH infections [26] and are targeted in mass deworming programs [27]. During the baseline study, the child’s infection status with STH and their hematocrit levels will be determined. A study conducted in 2008 in Cu Chi district found that hookworms were the predominant STH in this area, with most children experiencing low infection intensities [25]. Since heavy hookworm infections may lead to anemia [28], those children who are both positive for hookworms and anemic will be excluded from the subsequent clinical trial and will be treated with 400 mg albendazole and iron supplements. However, a recent Cochrane review and meta-analysis found no evidence of any effect of deworming on the average hemoglobin levels of children over 16 years of age in 45 randomized controlled trials [10].

### Inclusion of participants and informed consent

A letter of invitation will be sent home to all school children attending three primary schools in Cu Chi district in Ho Chi Minh City, Vietnam, inviting them to participate in this study. A meeting will be held at the schools so that parents can learn about the study. Here, study staff will describe the purpose of the study, the study procedures, possible risks/benefits, the rights and responsibilities of participants, and alternatives to enrollment. Parents/guardians will be given a participant information sheet that describes the study using the local and lay language. They will be asked if they understand the contents of the study and will be given the chance to ask questions. Once all questions have been answered satisfactorily, parents/guardians who are interested in enrolling their children in the study will be asked to sign an informed consent. In the consent form, they will be able to enroll their child in the baseline study only or in both the baseline study and the clinical trial. They will also consent to whether they agree to long-term storage of the collected samples for research purposes. The consent form will also explain to parents/guardians that they are free to withdraw their child from the study at any time and that information collected up to their child’s withdrawal will be retained. Children over 10 years of age will also be required to provide written assent in order to participate in this study. The study staff will also explain to all parents/guardians interested in enrolling their child in both the baseline study and the clinical trial that their child may or may not be randomly selected into the trial based upon the results from the baseline study. Parents/guardians will be informed whether or not their child is selected to participate in the clinical trial. If the parents, guardians, or children are indecisive about enrollment, they will be given up to 1 week to consider entry into the study, after which point the participant will no longer be eligible.

### Routine follow-up and sample collection

#### Baseline study

All enrolled children will receive a study identification (ID) number upon enrollment, which will be used to label any collected sample and to help ensure the confidentiality of the participant’s identity. The parent/guardian of the enrolled child will be asked to complete a short questionnaire regarding the child’s demographics, their daily habits, household information, and potential sources of infection. A study nurse will also collect information regarding the child’s weight and height. A study nurse will train the parent/guardian on stool collection and will provide them with a sterile stool collection pot. The child will provide one stool sample for helminth detection and for the detection of viral and bacterial pathogens that cause diarrhea, using real-time PCR and by the Kato-Katz method for microscopy. A finger-prick blood sample will also be collected to calculate hematocrit levels as a measure of anemia [29] (Fig. 1).

#### Clinical trial

From the baseline study, 350 helminth-infected and 350 helminth-uninfected children will be enrolled in the subsequent clinical trial. These consented participants will complete a short questionnaire regarding changes to their contact information, and a study nurse will measure their height and weight. Albendazole or placebo treatment will be administered once every 3 months for 12 months. At the 12-month time point, all participants will receive albendazole treatment. Stool samples will be collected once every 3 months (immediately before any treatment is administered) and 2 weeks after the first and third treatments to evaluate the efficacy of anthelmintic treatment in helminth removal and to quantify variation among individuals in their propensity to acquire helminth infections. Parents/guardians will be contacted weekly by phone to request information on whether their child has experienced any diarrheal episodes in the preceding 7 days. When a participant alerts the study nurse of a diarrheal episode, the study nurse will travel to the individual’s home to collect a stool sample and to administer a short questionnaire regarding the participant’s diarrheal symptoms and duration. A sterile stool collection pot will also be left with the parents/guardians for a stool sample collection 2 weeks after the last diarrheal episode. Four milliliters of venous blood will also be collected by the study nurse once every 6 months to measure hematocrit levels and any changes to immunity with helminth infection and removal over time. A finger-prick blood sample will also be collected in the third and the ninth month of the study to measure hematocrit levels in all children (Fig. 1).

### Data management

A case report form (CRF) will be used to collect data for each participant. Paper CRFs will be used and then entered into a password-protected, electronic database. A central database has been developed to ensure secure and confidential data management for this study. All paper records will be stored securely as per the Oxford University Clinical Research Unit (OUCRU) and the Vietnamese Ministry of Health guidelines and will be accessible to trial staff and authorized personnel only. Participants will be identified only by their initials and study ID or randomization number on the CRF. Data entered into the electronic database will be reviewed and verified by a second study staff member to ensure accordance with paper records. For quality control, all required fields in a given section will need to be completed before progressing to subsequent sections, and entries from the CRF into the database will be limited to the options on the questionnaire through pull-down menus, in order to prevent the input of incorrect text. All electronic devices for data entry will be password-protected and accessible only by authorized users.

### Study medication and quality control

Albendazole is a benzimidazole anthelmintic that is effective against a wide range of intestinal helminths and is currently used in many mass deworming programs under WHO guidelines [30]. The 400 mg albendazole used in this study will be purchased from Imexpharm Pharmaceutical Joint Stock Corporation (Cao Lanh, Vietnam) and will be manufactured under the WHO good manufacturing process guidelines. Before the start of the study, a random subset of albendazole and placebo pills will be sent to an independent testing company to measure the quantities of ingredients in the medications to ensure that the drugs meet international quality standards. Sachets with the active drug will contain one chewable 400 mg albendazole pill (for easier consumption). Matching placebo pills will lack the active ingredient, albendazole sulfoxide, but will otherwise contain the same ingredients as the albendazole pill. All treatments will look and taste alike. All treatments will remain at the OUCRU with the study pharmacist and stored according to manufacturer’s instructions until use. Every 3 months, the medication will be securely transported to Cu Chi district, and a trained study nurse will administer the medication to the participants with water, based upon the participant’s study ID and randomization code found on each medication sachet.

### Treatment groups and duration of treatment

From the baseline study, 350 helminth-infected and 350 helminth-uninfected children will be randomized to 400 mg albendazole or placebo treatment in a 1:1 ratio with equal randomization between helminth-positive and helminth-negative individuals. The regimes will be identical in both study groups, namely doses once every 3 months for the first 9 months. At the 12-month time point, all participants will receive albendazole treatment. For each dose, one sachet of chewable albendazole or placebo treatment will be administered orally with water to the enrolled participants by a trained study nurse.

### Blinding and randomization

The trial participants, care providers, study investigators, outcome assessors, and data analysts will remain blinded throughout the trial period of 12 months. Treatment allocations will not be disclosed to the enrolled participants or study personnel until the conclusion of the study or in case of emergency. Only the study pharmacist, who is not involved in field or laboratory studies, will be privy to the treatment allocations in order to prepare the treatment packages accordingly. Block randomization using a 1:1 randomization ratio, stratification by helminth-infection status (infected versus uninfected), and variable block sizes of 4 and 6, respectively, will be used to randomize subjects into either the anthelmintic or placebo group. The randomization list will be generated according to OUCRU standard operating procedures. Briefly, a research biostatistician will establish a statistical code to generate the randomization list and transfer it to the study pharmacist. The study pharmacist will change the random seed, i.e., the initialization of the random number generator, in the statistical code in order to blind the research biostatistician and then run the code to prepare the final randomization list for treatment preparation. The randomization list will be password-protected and stored on a secure server to which only the study pharmacist has access. Randomization numbers, which correspond to a study ID number and a sachet of study medication, will be assigned to each enrolled participant in the clinical trial in a strict numerical sequence with individual school identifiers. Randomization numbers will be recorded on each participant’s CRF and used to identify the participant’s corresponding blinded treatment package. A total of four masked sachets containing either anthelmintic or placebo treatment will be prepared for each enrolled participant throughout the clinical trial. All participants will receive albendazole treatment at the conclusion of the study (month 12).

### Laboratory methods

All samples from the baseline study will be labeled with a study ID number, and all samples from the clinical trial will be labeled with a study ID and randomization number to ensure anonymity. Samples will be stored at 4 °C upon collection. At the end of each sampling day, all samples will be transported to the OUCRU laboratory in Ho Chi Minh City, Vietnam, aliquoted, and transferred to a freezer at −80 °C until further analyses.

#### Parasite detection in stool samples

Nucleic acids will be extracted from fecal samples using the MP FastDNA SPIN Kit for Soil, the NucliSENS easyMAG system, and/or the Roche MagNA pure 96 automated nucleic acid extraction machine. The extracted deoxyribonucleic acid (DNA) and ribonucleic acid (RNA) from all collected stool samples will be used as templates for real-time PCR for helminth, bacteria, and virus identification. Samples will be tested for *A. lumbricoides*, *T. trichiura*, *N. americanus*, *A. duodenale*, and *Strongyloides stercoralis* for helminth infection, *Shigella*, *Campylobacter*, and *Salmonella* for bacterial infection, and Rotavirus and Norovirus for viral infections using previously validated primers and probes [31–33]. Fecal samples will also undergo microscopic investigation using the Kato-Katz method for helminth detection and egg quantification during the baseline study. Diarrheal stool samples will be analyzed using the Luminex xTAG Gastrointestinal Pathogen Panel for simultaneous detection and identification of a wide array of intestinal pathogens. Additionally, stool samples will be stored and assayed retrospectively, where required, to detect additional pathogens.

#### Determination of anemia in blood samples

Blood samples will be collected alternately via finger-prick or venous draws to monitor hematocrit levels and anemia in children for the baseline study and clinical trial. Blood for anemia measures will be collected in capillary tubes and spun in a microhematocrit centrifuge. The volume of red blood cells compared to the total blood volume (red blood cells and plasma) will be measured using a manual microhematocrit reader. Anemia will be defined using age-specific WHO thresholds adjusted on the basis of altitude. According to the WHO, children aged 5–11 years who have a hematocrit less than 0.34 and children aged 12–15 years who have a hematocrit less than 0.36, are considered anemic [29].

#### Immune markers in venous blood samples

For the clinical trial, 20 % of enrolled participants will have their venous blood samples separated into plasma, peripheral blood mononuclear cells (PBMCs), and red blood cells by density-gradient centrifugation at each sample collection. A random number generator will be used to equally subsample from the participants in each arm of the study, and the samples from these participants will be processed for PBMCs at each venous blood draw. The plasma will be removed and aliquoted for storage at −20 °C for antibody detection against parasite-specific antigens. PBMCs will be stimulated and cultured at 37 °C for 48 hours for cytokine production. The supernatants will be collected and stored at −20 °C until assayed for cytokine content (e.g., production of interferon gamma (IFNγ), tumor necrosis factor alpha (TNFα), interleukin (IL)-4, IL-13, IL-10, IL-17, IL-22) using bead-based immunoassays. Blood from the remaining children will be centrifuged and separated into plasma and cells upon collection. The plasma will be removed and aliquoted for storage at −20 °C for cytokine detection and for antibody detection against parasite-specific antigens by enzyme-linked immunosorbent assay.

#### Microbiota analyses by 16 s sequencing

DNA extracted from stool samples will be used for high-throughput Illumina sequencing to generate sequence data on the taxonomic composition of the gut microbiota. Sequences will be assigned to operational taxonomic units (OTUs), and the relative abundance of microbial taxa will be calculated using the proportion of OTUs present in each sample. Tests to identify significant differences in the relative abundance of bacterial taxa between groups will be conducted, and *α*-diversity and *β-*diversity measurements will be made. Statistical methods, combining dimension reduction using principal coordinate analysis and regression techniques for correlating microbiota analysis and parasite identification with immune data from blood, will be used to generate a comprehensive portrait of cellular and molecular events in the intestinal tract.

### Endpoints

#### Primary endpoint

The primary endpoint of this study is the total incidence of diarrheal disease, i.e., the total number of diarrheal episodes assessed through weekly active and passive surveillance during the 12-month study period. Diarrhea will be defined according to WHO guidelines, which is three or more loose stools in a 24-hour period or at least one bloody/mucoid stool [34]. To be considered a new episode of diarrhea, at least three intervening days of normal stools without other gastrointestinal symptoms need to have passed between diarrheal occurrences [35].

#### Secondary endpoints

The secondary endpoints are:

1. Prevalence and intensity of soil-transmitted helminth infections at 0, 0.5, 3, 6, 6.5, 9, and 12 months, and during and 2 weeks after cases of diarrhea
2. Prevalence and intensity of enteric viral and bacterial infections that cause diarrhea at 0, 3, 6, 9, and 12 months, and during and 2 weeks after cases of diarrhea
3. Changes in fecal microbiota composition at 0, 0.5, 3, 6, 9, and 12 months, and during and 2 weeks after cases of diarrhea
4. Changes in blood cytokine levels (TH1, TH2, TH17, and Treg) at 0, 6, and 12 months
5. Antibody isotype responses to helminth and diarrheal antigens at 0, 6, and 12 months
6. Mean *z* scores (height-for-age, weight-for-age, weight-for-height) at 0 and 12 months
7. Number and severity of adverse events (AE) during the 12-month study period

### Sample size and statistical considerations

In southern Vietnam, approximately 30 % of school-aged children are infected with at least one species of STH [20]. This percentage was supported by our preliminary study of helminth infections in healthy children in Ho Chi Minh City, Vietnam (our unpublished data). Most data on diarrheal incidences due to enteric infections have focused on children under 5 years of age. For the bacterial pathogens, *Shigella*, *Campylobacter*, and *Escherichia coli*, diarrheal rates in this age group range from 3.3 cases/child per year in children under 1 year old to 0.7 cases/child per year in 4-year-olds in Vietnam [36]. Few studies have investigated the incidence of diarrheal events in children over 5 years of age. Considering the diarrheal attack rate in 4-year-olds due to these three pathogens, it is estimated that school children over 5 years of age will have a probability of at least 0.6 of experiencing one or more diarrheal episodes due to any viral or bacterial species during the 12-month follow-up duration. The clinical trial is powered to detect a 15 % absolute increase of this probability (from 0.6 to 0.75) due to anthelmintic treatment in both the subgroups of helminth-infected and helminth-uninfected participants. For 80 % power at the two-sided 5 % significance level, a total of 298 helminth-infected and 298 helminth-uninfected participants will be needed. Of note, this sample size calculation is conservative insofar that the actual study endpoint measures the number of diarrheal episodes and is thus more sensitive than the binary assessment of whether at least one episode occurred or not. To account for potential inadequacies in our assumptions and some loss to follow-up, the sample size was increased by 15 %. Thus, a total sample size of 700 participants: 350 helminth-infected and 350 helminth-uninfected children, will be recruited from the baseline study (using a random number generator to subsample from those groups, as needed) to the clinical trial and will be equally randomized to receive either anthelmintic or placebo treatment. Assuming that approximately 30 % of school-aged children are infected with at least one species of STH infection [20], to enroll 350 helminth-infected children, up to 1800 school children will be enrolled in the baseline study, in order to account for any uncertainties in the helminth prevalence during the baseline study and for decreased participant enrollment for the clinical trial.

### Statistical analysis

The main analysis population includes all randomized participants, and the analysis will be conducted according to the randomized treatment arm following the intention-to-treat principle. Subgroup analyses according to helminth infection status at enrollment (infected versus uninfected) will also be performed for all endpoints.

The primary endpoint, the number of diarrheal episodes per subject, will be compared between the two study arms based on mixed-effects Poisson regression models, with treatment as the main covariate and adjustment for helminth infection status at enrollment (infected versus uninfected). To account for subjects lost to follow-up, the subject’s (log-transformed) follow-up duration will be included as an offset in the Poisson regression model. Moreover, to account for potential heterogeneity (i.e., larger between-participant variability than expected by the Poisson regression model), the participant variable will be included as a random effect in the model. If necessary, a random effect of family or household will also be included to account for non-independence of siblings or other co-inhabitants of a household. In a second step, the effect of the following covariates on diarrheal incidence will be explored: age, sex, body mass index, and household characteristics (e.g., type of water supply). A random effect of individual will be included to account for non-independence of repeated diarrheal episodes for a given individual. Additionally, the effect of helminth infection and helminth infection intensity, as determined from longitudinal stool samples, on diarrheal incidence will be investigated.

For the secondary endpoints, the proportion of participants with STH and enteric viral and bacterial infections, as well as the infection intensities at each time point, will be summarized and visualized as line-plots over time. Formal comparisons between the treatment arms will be based on logistic mixed-effects models for proportions and linear mixed-effects models for (log-transformed) intensities using the same covariates and random effects as for the primary endpoint. The baseline measurements will be included in the analysis, but modeled as not depending on treatment arm. For the main comparison between treatment arms, only stool samples from scheduled visits will be included, as stool samples collected 2 weeks after diarrheal episodes may be unequally distributed between treatment arms. However, these additional stool samples will be included in the exploratory analyses. The effect of additional household and environmental factors present at baseline on helminth, viral, and bacterial infections will also be investigated using the same statistical modeling approach outlined above.

The taxonomic composition of the gut microbiota will be analyzed before and after helminth infection, anthelmintic treatment, and diarrhea and will be compared with individual baseline levels, between the two treatment arms, and between helminth-infected and helminth-uninfected individuals. The mothur pipeline [37] will be used to detect microbial composition and diversity between and within samples based upon the proportion of OTUs in each sample and to apply appropriate corrections for any differences driven by rare taxonomic groups of microbes. Tests, such as the LDA Effect Size (LEfSe) algorithm [38], will be used to identify significant differences in bacterial taxa between the different groups of interest.

Cytokine levels and antibody profiles in response to parasite-specific antigens will be analyzed from longitudinally collected blood samples. Cytokine and antibody data will be log-transformed for all analyses, and linear mixed-effects modeling for analysis will be conducted as outline above. Additionally, changes in height-for-age, weight-for-age, and weight-for-height *z* scores between baseline and 12 months will be compared between the two treatment arms based on linear regression with adjustment for the baseline *z* score. Random effects to account for non-independence of siblings or other co-inhabitants from the same household will be included if necessary. Additionally, the overall number of participants with at least one adverse event (AE) or serious adverse event (SAE), respectively, will be compared between the two study arms using Fisher’s exact test. Descriptive tables will be used to summarize the frequency of all recorded AEs and SAEs by arm.

### Reporting of serious adverse events

All grade three to grade five AEs and SAEs will be recorded on the participant’s CRF. AEs are graded according to their severity from 0 to 5 according to the Common Terminology Criteria for Adverse Events from National Cancer Institute [39]. A study physician will use clinical judgment to assess whether the AE/SAE is drug and/or study-related, or if it is due to the natural history of the underlying condition or disease. If a participant experiences a SAE, the study physician will inform the principal investigator and complete the specific documentations and the CRF. All SAEs will be reported to the Institutional Review Boards (IRBs) of reference, sponsors, and the Data and Safety Monitoring Board for this study.

### Data monitoring

The study will be overseen by an independent Data and Safety Monitoring Board (DSMB), who will review unblinded study data at bi-annual safety interim analyses, once before the initiation of the study and once at the 6-month time point, and can recommend stopping or modification of the trial in case accruing evidence suggests that continuing the study would be unethical. The constituents of the DSMB will have no role in the planning, conduct, or implementation of the trial itself and will serve only as a third party internal review of data and safety. The DSMB may recommend termination or modification of the study if preliminary data indicate beyond a reasonable doubt that anthelmintic treatment increases the total incidence of diarrheal disease (primary endpoint) or if the observed diarrheal incidence in either treatment group is at least tripled compared to the other. The Haybittle-Peto boundary, requiring a *p* value <0.001 at interim analysis to consider stopping for superiority of placebo, will be used as a guidance. The DSMB may also recommend termination or modification if preliminary data clearly suggest that withholding anthelmintic treatment (i.e., the placebo group) is harmful in terms of anemia. A less conservative *p* value <0.01 in the direction of harm for either of these endpoints will be used as guidance. The DSMB recommendation should not be based purely on statistical tables and *p* values, but also requires clinical judgment.

## Discussion

The WHO plans for the regular administration of anthelmintics to 75 % of all school-aged children at risk of helminth-induced morbidity by 2020 [40]. Given the rapid expansion of single-dose mass administration of anthelmintic therapy at a global level, studies assessing the impact of anthelmintic treatments on other pathogens are needed. Recently, anthelmintic trials have begun to monitor for altered risks of malarial infections in dewormed individuals [41–43]. However, studies investigating the impact of anthelmintic treatments on pathogens other than the targeted helminths remain rare. Here, we plan to investigate whether anthelmintic treatments alter the incidence of diarrheal disease caused by viral and bacterial pathogens in school children in southern Vietnam.

Two types of single-dose anthelmintics, 400 mg albendazole and 500 mg mebendazole, are recommended by the WHO for STH control [30]. For STH, the efficacy of albendazole and mebendazole varies depending on helminth species. The cure rates after a single dose of 400 mg albendazole are around 96 % for *A. lumbricoides*, 34 % for *T. trichiura*, and 69 % for hookworms [44]. With a single dose of 500 mg mebendazole, the cure rates for infection by *A. lumbricoides*, *T. trichiura*, and hookworms are around 93 %, 40 % and 31 %, respectively. Due to the higher cure rate of albendazole for hookworm infections, which are the predominant STH infections in Cu Chi district [25], and to follow the previous medication used in school-based deworming programs in this area, 400 mg albendazole was selected to be used in this study. As part of the clinical trial, we will also collect stool samples 2 weeks after the first and third treatments, which will allow us to further investigate the efficacy of albendazole in reducing STH burdens in Cu Chi district. Irrespective of the trial outcome, the data generated from this study will generate valuable information regarding the rates of helminth acquisition, intensities of helminth infections, incidence of diarrhea, and efficacy of anthelmintic treatments in school-aged children in Cu Chi district, which can inform Vietnam health officials when designing future public health programs.

Re-infection occurs rapidly after all types of deworming. Within 3 months post treatment, approximately one third of treated individuals are re-infected with STH and more than 50 % of individuals are typically re-infected 6 months post treatment [45]. In order to decrease the chances of re-infection in the anthelmintic arm, we will administer anthelmintic and placebo treatments once every 3 months in contrast to current school-based deworming programs in Vietnam, which administer anthelmintic treatments once every 6 months. The increased frequency of anthelmintic administration poses no known health risk to the individual [46]. This study design will help to minimize the helminth burden in those individuals in the anthelmintic arm and thus increase the chances of observing any potential effect of anthelmintics on diarrheal incidence.

As it is difficult to accurately assess the burden of diarrheal disease [47], an important strength of this study is the active surveillance of diarrheal cases that will be conducted during the trial. We will monitor for incidences of diarrhea through weekly active and passive surveillance. A study nurse will call the parents/guardians of each participant weekly, asking if any incidence of diarrhea has occurred. Participants will also inform the study nurse of diarrheal cases through passive surveillance. We will also collect stool samples and information regarding diarrheal symptoms with each diarrheal episode. Most studies on diarrhea focus on children under 5 years of age, and there is currently a dearth of studies reporting on diarrhea in school-aged children [48]. This study will provide valuable information regarding the causative agents and frequency of diarrhea in this age group in Cu Chi district of Ho Chi Minh City, Vietnam.

While the primary endpoint of the study is the incidence of diarrheal disease assessed by 12 months, the sample collections in this trial will also help to disentangle potential mechanisms by which helminths may be altering the incidence of viral- and bacteria-induced diarrhea. Helminths can affect both the activation of the human immune system and the composition of the gut microbiota (e.g., [49, 50]), and these alterations may impact the colonization or invasion of enteric viral and bacterial pathogens that share the same intestinal niche. The blood and stool samples collected in this study will help to assess biological differences in immune function and gut microbial communities between infected and uninfected individuals and between treatment groups, which may elucidate potential mechanisms by which helminths interact with diarrheal pathogens in the human gut.

Ultimately, this study will provide an important first test of whether anthelmintic treatment has unintended effects on the health of school children and their subsequent risks of infections due to enteric viral and bacterial pathogens. This study is timely and much needed, as diarrhea remains a major concern for child health worldwide, as deworming programs are becoming routine in developing countries, and as growing experimental evidence suggests that anthelmintic treatments may increase host susceptibility to other infections (bacteria, viruses, or protozoa) [14–18]. We aim to investigate whether current mass administrations of anthelmintic treatments indirectly impact the disease dynamics of co-infecting pathogens by focusing on diarrheal pathogens that inhabit the same intestinal niche as STH. Our results will provide new insights into the role of co-infections in the success of treatment strategies in developing countries and will elucidate the importance of both the microbiota and macrobiota in human health. Further relevant aims of this study are to inform public health policies of potential indirect effects of mass deworming programs on other co-infecting pathogens and to inform WHO recommendations on anthelmintic dose regimens for Cu Chi district and beyond.

## Trial status

This study has started participant recruitment.

## Abbreviations

AE, adverse event; CRF, case report form; DSMB, Data and Safety Monitoring Board; ID, identification; IL, interleukin; IRB, Institutional Review Board; LEfSe, LDA Effect Size; mg, milligrams; OTUs, operational taxonomic units; OUCRU, Oxford University Clinical Research Unit; PBMCs, peripheral blood mononuclear cells; PCR, polymerase chain reaction; SAE, serious adverse event; STH, soil-transmitted helminth; WHO, World Health Organization

## Additional file

Additional file 1: SPIRIT 2013: SPIRIT (Standard Protocol Items: Recommendations for Interventional Trials) checklist for clinical trial protocols. (DOC 120 kb)
